# Causal links between sedentary behavior, physical activity, and psychiatric disorders: a Mendelian randomization study

**DOI:** 10.1186/s12991-024-00495-0

**Published:** 2024-02-29

**Authors:** Hongjun Ba, Lili Zhang, Huimin Peng, Xiufang He, Yao Wang

**Affiliations:** 1 Department of Pediatric Cardiology, Heart Center, First Affiliated Hospital of Sun Yat-sen University, 58# Zhongshan Road 2, Guangzhou, 510080 China; 2 Key Laboratory on Assisted Circulation, Ministry of Health, 58# Zhongshan Road 2, Guangzhou, 510080 China; 3 Cancer Hospital, Guangzhou Medical University, Guangzhou, 510095 China

**Keywords:** Psychiatric disorder, Sedentary behavior, Physical activity, Mendelian randomized study, Causal relationship

## Abstract

**Background:**

Studies suggest a correlation between excessive sedentary behavior, insufficient physical activity, and an elevated likelihood of experiencing psychiatric disorder. Nonetheless, the precise influence of sedentary behavior and physical activity on psychiatric disorder remains uncertain. Hence, the objective of this research was to investigate the possible causal relationship between sedentary behavior, physical activity, and the susceptibility to psychiatric disorder (depression, schizophrenia and bipolar disorder), utilizing a two-sample Mendelian randomization (MR) approach.

**Methods:**

Potential genetic instruments related to sedentary leisure behaviors were identified from the UK Biobank database, specifically a summary-level genome-wide association study (GWAS) involving 422,218 individuals of European descent. The UK Biobank database also provided the GWAS data for physical activity. Primary analysis was performed using inverse variance weighting (IVW) to assess the causal relationship between sedentary behavior, physical activity, and the risk of psychiatric disorder (depression, schizophrenia and bipolar disorder). Sensitivity analysis was conducted using Cochran’s Q test, the MR–Egger intercept test, the MR-pleiotropy RESidual sum and outlier test, leave-one-out analysis, and funnel plot analysis.

**Results:**

According to the IVW analysis, there was a significant association between genetically predicted leisure television watching and an increased risk of depression (odds ratio [OR] = 1.027, 95% confidence interval [CI]: 1.001–1.053; *P* = 0.04). The IVW analysis also indicated that there was a decreased risk of depression associated with fraction accelerations of > 425 milligravities, as measured by accelerometers (OR = 0.951, 95%CI: 0.914–0.989; *P* = 0.013). The other MR methods obtained consistent but non-significant results in the same direction. However, there was no evidence of a causal association between genetic liability for moderate-to-vigorous physical activity, accelerometer-assessed physical activity, computer use, or driving and the risk of depression. Furthermore, IVW analysis has also found that driving has a slight effect in reducing the risk of schizophrenia (OR = 0.092, 95%CI: 0.010–0.827; *P* = 0.033), while leisure television viewing has a significant protective effect against the onset of bipolar disorder (OR = 0.719, 95%CI: 0.567–0.912; *P* = 0.006).

**Conclusion:**

The study provides compelling evidence of a link between depression, bipolar disorder, and excessive TV watching. Furthermore, it suggests that higher accelerometer-assessed fraction accelerations of > 425 milligravities can serve as a genetic protective factor against depression. To mitigate the risk of developing depression, it is advisable to reduce sedentary activities, particularly television watching, and prioritize engaging in vigorous physical exercise.

**Supplementary Information:**

The online version contains supplementary material available at 10.1186/s12991-024-00495-0.

## Background

Psychiatric disorders are widespread mental health problems that affect a significant proportion of the global population. It is estimated that these disorders impact approximately 22.1% of the world’s population [1]. The origins of psychiatric disorders are complex, involving a combination of genetic, environmental, and neurobiological factors.

Recent research has also explored the role of leisure sedentary behavior and physical activity in the development of psychiatric disorders. Evidence indicates that excessive leisure sedentary behavior is associated with an increased risk of developing depression [2]. In fact, leisure sedentary behavior can lead to physiological changes, decreased exposure to natural light, and reduced social interaction, all of which may contribute to the severity and development of depressive symptoms [3]. Observational studies have also found a link between sedentary behavior and schizophrenia and bipolar disorder [4, 5]. Regular physical activity has been proven to have positive effects on mental health, including the alleviation of depressive symptoms [6]. Exercise boosts the levels of endorphins, serotonin, and other neurochemicals linked to improved mood and overall well-being [7, 8]. Nevertheless, there is still a lack of direct evidence regarding the causal influence of leisure sedentary behavior and physical activity on psychiatric disorders.

Mendelian randomization (MR) analysis is a powerful tool for examining the causal connections between risk factors and diseases. By using genetic variations as the instrumental variables (IVs), MR provides more reliable evidence of causality compared with traditional observational studies [9]. A notable advantage of MR is its ability to overcome the common limitations encountered in observational studies, such as confounding and reverse causality. By utilizing genetically determined variations at conception, MR can provide stronger evidence of causality since these variations are less influenced by external factors that may be linked to both the risk factors and the disease outcomes. The impact of MR in investigating causality has been extensively explored across a range of diseases, including prostate cancer [10], inflammatory bowel disease [11], cardiovascular diseases [12], and gastrointestinal diseases [13], as well as their association with depression. Nonetheless, the connection between psychiatric disorder and leisure sedentary behavior and physical activity has not been thoroughly explored. Consequently, a comprehensive analysis was undertaken using a two-sample MR approach and incorporated data from genome-wide association studies (GWAS) to delve deeper into the causal associations between leisure sedentary behavior, physical activity, and psychiatric disorders (depression, schizophrenia and bipolar disorder).

## Materials and methods

### Study design

**Fig. 1:**
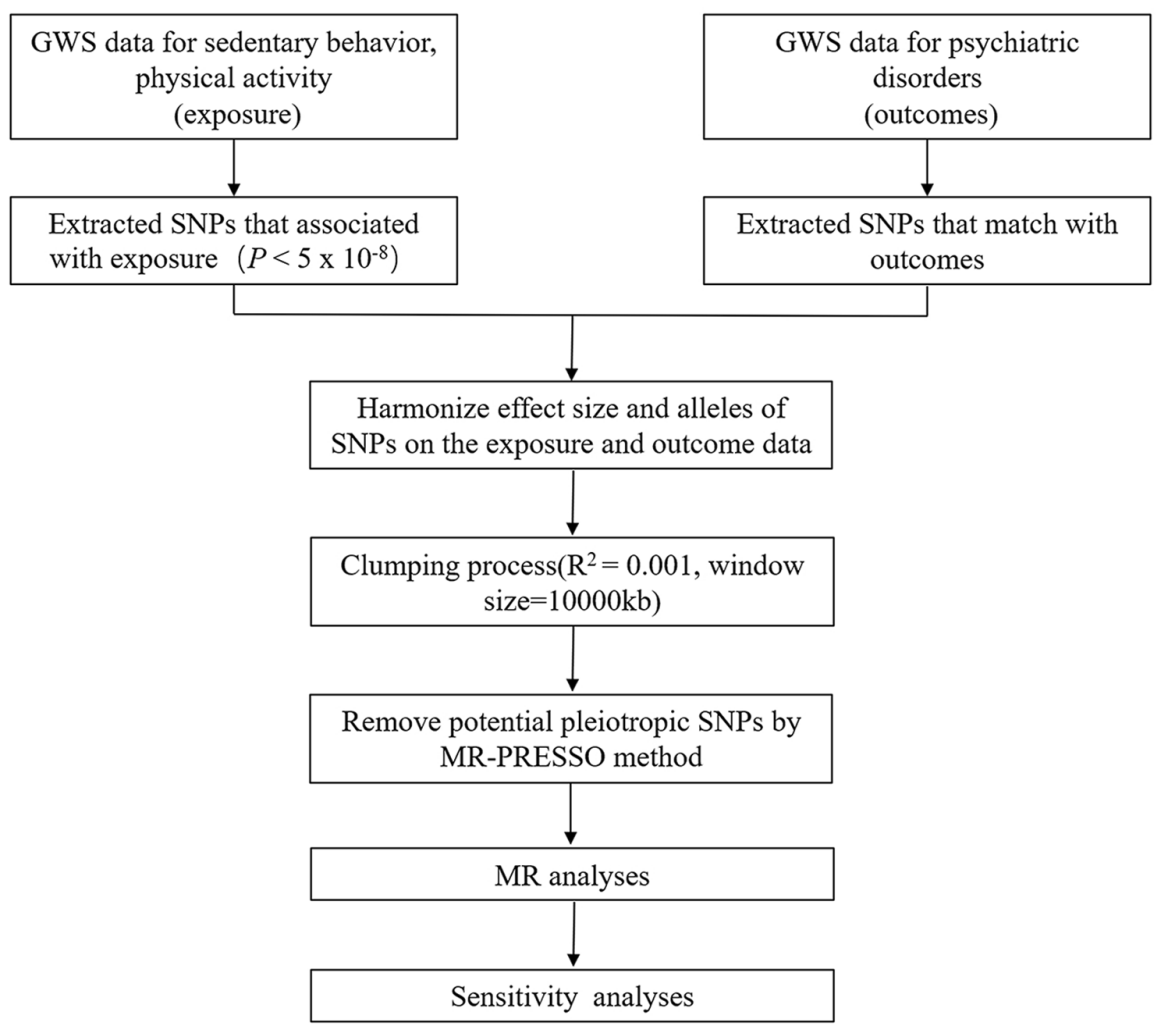
Study design and workflow. Note: Outcomes including depression, schizophrenia, and bipolar disorder

A MR study was carried out utilizing two samples to assess the causal relationship between leisure sedentary behavior, physical activity, and psychiatric disorders (depression, schizophrenia and bipolar disorder) (as depicted in Fig. 1). Psychiatric disorders mainly include depression, schizophrenia and bipolar disorder. To accomplish this, the MR method relied on three critical assumptions: [1] the genetic instrumental variables employed in the analysis exhibited a robust association with sedentary behavior and physical activity; [2] the genetic instrumental variables remained unaffected by any confounding variables; and [3] the genetic instrumental variables were solely connected to the outcomes through sedentary behavior and physical activity, without any involvement of other causal pathways [14]. The data primarily originated from independent GWAS data sources.

### Data sources and instrumental variables selection

#### Exposures in genome-wide association studies: leisure sedentary behaviors and physical activity

The genetic instruments considered for leisure sedentary behavior were determined by analyzing data from the most recent summary-level GWAS. This research involved 422,218 individuals of European descent who were part of the UK Biobank [15]. Within this GWAS, three main categories of sedentary behaviors linked to participation in leisure activities were considered: watching TV, using a computer, and driving. The participants were asked to provide information regarding their daily time allocation to different activities through responding to the following inquiries: “What is the typical duration of time you spend per day watching television?” “How many hours do you usually use a computer within a day? (excluding computer use at work)”, and “On average, how much time do you spend driving in a day?”. According to the gathered responses, the participants dedicated a daily average of 2.8 h to watching television, with a standard deviation (SD) of 1.5 h. Similarly, computer usage accounted for approximately 1.0 h per day, with a SD of 1.2 h, while driving averaged 0.9 h per day, with a SD of 1.0 h. Considering multiple variables, including age, gender, smoking habits, hypertension, diabetes, body mass index (BMI), Townsend deprivation index, levels of physical activity, weekly alcohol intake, and educational background, the study identified a total of 209 single nucleotide polymorphisms (SNPs) associated with watching television, 35 SNPs associated with computer usage, and four SNPs associated with driving (*P* < 1 × 10^–8^) [15].

To ensure the quality of the SNP data for the MR analysis, rigorous filtering procedures were implemented. Initially, we combined SNPs that were in linkage disequilibrium within a 10-mb range, with an R^2^ value of > 0.001. To evaluate the effectiveness of the genetic instruments, SNPs with an *F* statistic of < 10 were excluded in the subsequent analysis. Additionally, SNPs were associated with the corresponding exposure using a genome-wide significance threshold of *P* < 5 × 10^− 8^. To eliminate any ambiguity, palindromic SNPs were also removed through harmonizing processes. Furthermore, any potential pleiotropy was addressed by employing the MR-pleiotropy RESidual sum and outlier method to remove any SNPs before MR analysis was re-performed to ensure robustness. Ultimately, 78 SNPs were employed as IVs for television viewing, 15 SNPs for computer usage, and one SNP for driving. For more comprehensive details regarding the SNPs utilized as IVs, refer to Additional file 1: Tables S1–S3.

The GWAS data for physical activity were provided by the UK Biobank [16, 17]. Adjustments in the data included age, sex, the first 10 genomic principal components from the genotyping chip, the center, the season (month) of the center visit, and the use of an accelerometer. Physical activity was measured using three different measures: self-reported levels of moderate-to-vigorous physical activity (MVPA), objectively measured physical activity using accelerometers, and accelerometer-based percentage accelerations exceeding 425 milligravities. Comprehensive details of these physical activity measures can be found in the original study [16]. To identify IVs for physical activity, the following criteria were applied [18]: [1] SNPs with genome-wide significance (*P* < 5 × 10^–8^); [2] SNP clustering using the PLINK algorithm (r^2^ threshold = 0.001 and window size = 10 MB); and [3] exclusion of SNPs that may have pleiotropic effects. Consequently, the investigation employed 14 SNPs for MVPA, seven SNPs for accelerometer-measured physical activity, and seven SNPs for accelerations exceeding 425 milligravities as the IVs (Additional file 1: Tables S4–S6).

### Outcomes in genome-wide association studies

Summary statistics were obtained from publicly available large-scale GWAS on depression [19]. Depression was based on self-reported help-seeking behaviour for mental health difficulties from either a general practitioner or psychiatrist. To ensure the exclusion of individuals potentially impacted by conditions such as schizophrenia, a thorough screening procedure was implemented. This procedure involved the utilization of self-reported information, responses generated by the touchscreen, or ICD codes obtained from medical facility admission records. In addition, information was obtained from individuals who disclosed the usage of antipsychotic medication during a spoken interview. Conversely, control participants were excluded if they reported taking antidepressant medication, had a documented diagnosis of a mood disorder with depressive symptoms in their medical facility admission records, or self-reported experiencing depressive feelings. This study was carried out with the explicit endorsement of the NHS National Research Ethics Service, and all participants voluntarily provided informed written consent. To ascertain statistical significance, the threshold was established at *P* < 5 × 10^− 8^, adhering to the customary convention [19].

GWAS data for schizophrenia [20] and bipolar disorder [21] were obtained from the Psychiatric Genomics Consortium.

### Statistical analysis

#### Mendelian randomization analysis

In this two-sample MR study, the inverse-variance weighted (IVW) method was used as the primary analytical method to assess the causality between leisure sedentary behavior, physical activity, and depression. The IVW method involves the use of the Wald ratio method to calculate the exposure outcome effect for each SNP and weighted linear regression with a forced intercept equal to zero. If the IVs satisfy three fundamental assumptions, this method offers more precise estimation and testing capabilities. Various sensitivity analyses were conducted to tackle issues related to unknown and unmeasured confounding factors, including MR-Egger regression [22], weighted median estimator (WME) analysis [23], and simple mode [24], weighted mode, and outlier methods. Among these methods, WME provides consistent causal relationship estimates, even if up to 50% of genetic variation is invalid. Unlike other techniques, WME considers both potential linkage imbalances and sampling errors among SNPs when estimating the impact of IVs on exposure [24].

#### Sensitivity analyses

The *F* statistic technique was utilized to gauge the potency of the IVs for the selected SNPs in this study. A threshold *F*-value of > 10, which is commonly recommended in MR analytical studies, was used. The strength of each genetic tool was estimated using the *F* statistic: *F* = *R*^*2*^(*N* − 2)/(1 − *R*^*2*^), where *R*^*2*^ is equal to the proportion of the variance explained by the genetic instrument and *N* is the effective sample size of the SNP-micronutrient associated GWAS [25]. As an additional measure, Cochran’s Q test was employed to examine the heterogeneity among the SNPs, which was derived from IVW evaluations. Sensitivity analysis was performed using leave-one-out (LOO) analysis by removing one SNP to ensure that no single SNP had an escalating impact on the analysis. This was combined with IVW to assess the total impact of the remaining SNPS. The analysis was conducted using R software (version 4.3.0) and R software package two-sample MR. The results are presented as the mean effect of a 1-SD-increase in BMI, along with the corresponding 95% confidence interval (CI). A two-sided *P-*value of < 0.05 was regarded as statistically significant.

#### Risk factors

To explore the genetic associations between leisure sedentary behavior and physical activity in relation to depression, an investigation was conducted on various potential mediators. These mediators encompassed factors such as BMI, lipid levels (triglycerides and total cholesterol), type 2 diabetes (T2D), and fasting insulin levels. The genetic basis for BMI was determined using data from the UK Biobank, which included a cohort of 461,460 individuals of European ancestry. The dataset utilized for this analysis is publicly accessible through the MRC IEU OpenGWAS database and MR-Base project, with the GWAS-ID, ukb-b-19,953. To process this dataset, PHESANT-derived variables from the UK Biobank were employed.

Varied GWAS data from the UK Biobank [26] was employed to analyze the lipid profile. Information on genetic factors related to T2D was obtained from the Diabetes Genetics Replication and Meta-analysis (DIAGRAM) consortium [27]. Additionally, the Meta-Analyses of Glucose and Insulin-related traits Consortium (MAGIC) provided the GWAS data on fasting insulin [28]. Refer to Table 1 for comprehensive details on each data source.

**Table 1:** Data source of the depression-related risk factors

Traits	Consortium	Sample size	Ancestry	PubMed ID/GWAS- ID
Body mass index	MRC IEU	461,460	European	ukb-b-19953
Total cholesterol	UK Biobank	17,802	European	31217584
Triglycerides	UK Biobank	441,016	European	32203549
Type 2 diabetes	DIAGRAM	69,033	European	22885922
Fasting insulin	MAGIC	51,750	European	22581228

In this investigation, leisure sedentary behavior and physical activity were regarded as the exposures, while the aforementioned potential risk factors were employed as outcomes for the purpose of conducting MR. The primary findings were evaluated utilizing IVW estimates, with the significance level established at *P* ≤ 0.05.

## Results

### Mendelian randomization estimates

**Fig. 2:**
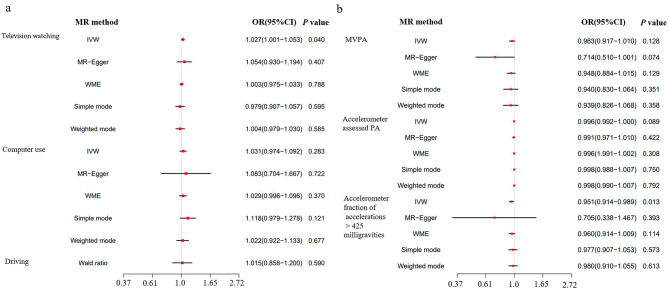
Causal effects of leisure sedentary behaviors and physical activity on depression. (**a**, **b**) Summary of the Mendelian randomization (MR) estimates derived from the inverse-variance weighted (IVW), weighted median (WM), and MR-Egger methods. MVPA: self-reported moderate-to-vigorous physical activity

The IVW analysis revealed a significant link between the anticipated genetic influence on leisure television viewing and an increased likelihood of experiencing depression (OR = 1.027, 95%CI: 1.001–1.053; *P* = 0.04) (Fig. 2a). Conversely, when evaluating the accelerometer data, it was observed that higher fraction accelerations exceeding 425 milligravities were associated with a reduced risk of depression (OR = 0.951, 95%CI: 0.914–0.989; *P* = 0.013) (Fig. 2b). However, no evidence of causation was found between genetic predisposition for MVPA, accelerometer-measured physical activity, computer utilization, or driving and the risk of depression (Fig. 2). Furthermore, while the results obtained through the other MR methods consistently aligned with the above findings, they failed to achieve statistical significance (Additional file 1: Table S7 and Fig. 2).

Furthermore, IVW analysis has also found that driving has a slight effect in reducing the risk of schizophrenia (OR = 0.092, 95%CI: 0.010–0.827; *P* = 0.033), while leisure television viewing has a significant protective effect against the onset of bipolar disorder (OR = 0.719, 95%CI: 0.567–0.912; *P* = 0.006). On the other hand, there is no apparent association between physical activity, computer use, and the onset of schizophrenia or bipolar disorder (Additional file 1: Table S8 and Table S9).

### Sensitivity analyses

**Fig. 3:**
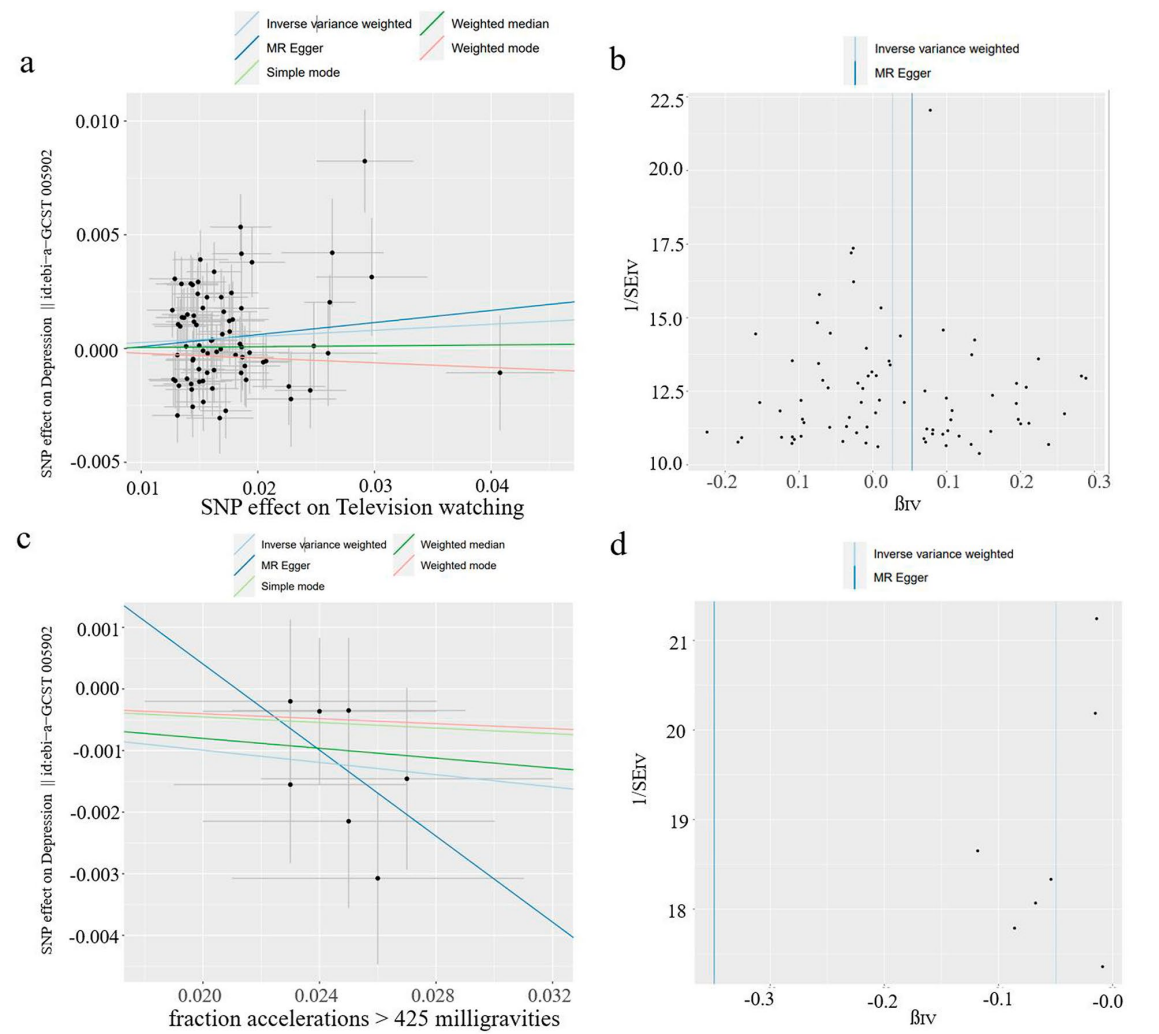
Scatter plots and funnel plots depicting the relationship between genetically predicted television watching and depression, and the relationship between genetically predicted accelerometer fraction accelerations of > 425 milligravities and depression. (**a**, **b**) Genetically predicted impact of television watching on depression; (**c**, **d**) genetically predicted impact of accelerometer fraction accelerations of > 425 milligravities on depression

**Fig. 4:**
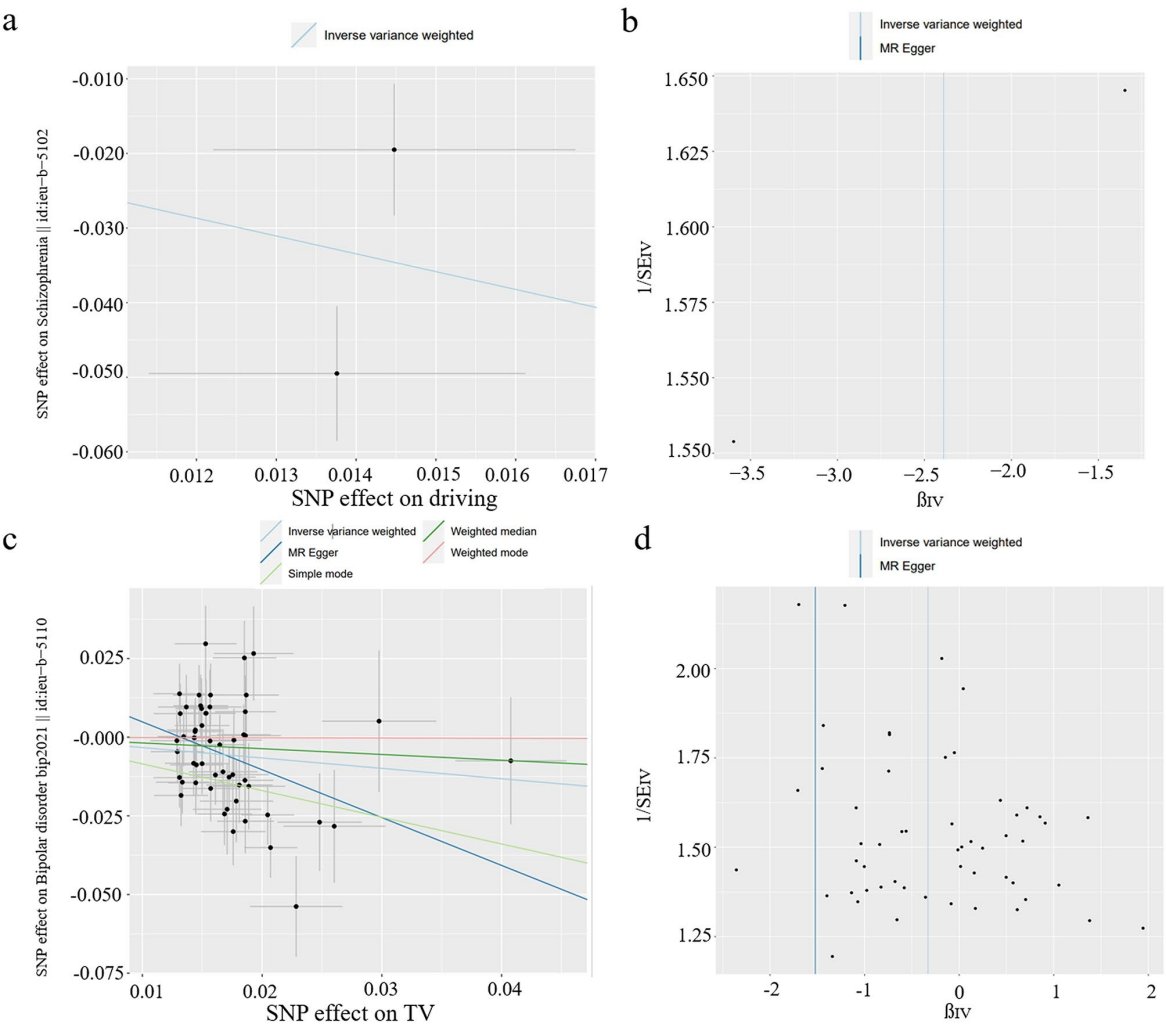
Scatter plots and funnel plots depicting the relationship between genetically predicted driving and schizophrenia, and the relationship between genetically predicted television watching and bipolar disorder. (**a**, **b**) Genetically predicted impact of driving on schizophrenia; (**c**, **d**) genetically predicted impact of television watching on bipolar disorder

To assess the robustness and reliability of the above findings, a variety of sensitivity analyses were conducted. These analyses included the use of different statistical tests, including Cochran’s Q test, the MR-Egger intercept test, and the MR-PRESSO global test (Table 2). The results of the MR-Egger intercept tests indicated that there was no evidence of horizontal pleiotropy, as all *P* values were > 0.05. However, when examining the Q test analysis for the relationship between television watching and depression (Q = 161.853, *P* = 5.53 × 10^− 8^), some heterogeneity was observed. It is important to note that this heterogeneity did not affect the validity of the MR estimates, as the random-effects IVW method used in this study helps to account for pooled heterogeneity. Furthermore, the absence of pleiotropy was confirmed by the Egger intercepts, which indicated that the MR estimates were not influenced by pleiotropic bias, even in the presence of heterogeneity (Figs. 3a and c and 4a and c). In the remaining analyses, no heterogeneity was observed. Moreover, the LOO analysis indicated that none of the SNPs had a significant impact on the results, while the funnel plots displayed a symmetrical distribution (Figs. 3b and d and 4b and d), suggesting that the estimates were not compromised (Additional file 2: Figures S1-S4).

**Table 2:** Sensitivity analysis of the causal association between leisure sedentary behaviors, physical activity and the risk of depression

Exposure	Outcome	Cochran Q test		MR‑Egger		MR‑PRESSO *P* value
		Q value	*p*	Intercept	*p*	
Television watching	depression	161.853	5.53 × 10^− 8^	0.0004	0.672	< 0.001
Computer use	depression	27.439	0.168	-0.0007	0.82	0.026
Driving	depression	-	-	-	-	-
MVPA	depression	14.633	0.331	0.004	0.105	0.332
Accelerometer assessed PA	depression	6.645	0.35	0.0013	0.623	0.392
Accelerometer fraction of accelerations > 425 milligravities	depression	3.725	0.713	0.007	0.458	0.732
Television watching	schizophrenia	154.939	6.50 × 10^− 10^	154.540	4.56 × 10^− 10^	< 0.001
Computer use	schizophrenia	34.156	0.002	32.672	0.002	0.003
Driving	schizophrenia	6.351	0.012	-	-	-
MVPA	schizophrenia	39.551	8.53 × 10^− 5^	38.648	6.08 × 10^− 5^	< 0.001
Accelerometer assessed PA	schizophrenia	9.238	0.099	8.827	0.066	0.137
Accelerometer fraction of accelerations > 425 milligravities	schizophrenia	11.440	0.076	7.256	0.202	0.091
Television watching	bipolar disorder	101.567	6.80 × 10^− 5^	94.474	2.89 × 10^− 4^	< 0.001
Computer use	bipolar disorder	16.918	0.261	14.383	0.347	0.273
Driving	bipolar disorder	14.394	0.002	13.522	0.001	-
MVPA	bipolar disorder	15.630	0.270	14.748	0.255	0.353
Accelerometer assessed PA	bipolar disorder	2.286	0.319	0.103	0.749	0.368
Accelerometer fraction of accelerations > 425 milligravities	bipolar disorder	0.088	0.957	0.038	0.845	0.755

MVPA: self-reported moderate to vigorous physical activity. PA: physical activity. - Driving has only one or two SNP

### Risk factor analysis

To examine the possible factors connecting an increased risk of depression with television watching, MR methods were utilized to analyze the attendant impact on various common risk factors associated with depression. As shown in Table 3, the results demonstrate the following findings. In the case of leisure sedentary behavior, a significant association was observed between a 1-SD-h increase (2.8 h) in television watching and a 0.479 higher BMI, a 99% increased risk of T2D, and a 0.168-pmol/L rise in fasting insulin levels.

**Table 3:** Risk factors analysis

Exposure	Outcomes	IVW		Heterogeneity		MREgger method	
		Causal effect (95% CI)	*p*	Q value	*p*	Intercept	*p*
Television watching	BMI	1.479(1.379–1.586)	5.28 × 10^− 28^	208.022	1.22 × 10^− 9^	0.0003	0.909
Television watching	Total cholesterol	1.024(0.864–1.214)	0.776	111.126	0.017	-0.0011	0.875
Television watching	Triglycerides	1.240(1.185–1.297)	6.86 × 10^− 21^	120.072	1.973 × 10^− 5^	0.002	1.997
Television watching	Type 2 diabetes	1.990(1.532–2.585)	2.45 × 10^− 7^	14.706	0.326	-0.0084	0.451
Television watching	Fasting insulin	1.168(1.092–1.250)	5.88 × 10^− 6^	84.211	0.011	0.004	0.162
fraction accelerations > 425 milligravities	BMI	0.780(0.687–0.885)	0.0001	10.238	0.036	-0.003	0.917
fraction accelerations > 425 milligravities	Total cholesterol	0.891(0.640–1.241)	0.495	5.442	0.488	0.0426	0.596
fraction accelerations > 425 milligravities	Triglycerides	0.846(0.761–0.940)	0.001	15.164	0.019	-0.001	0.952
fraction accelerations > 425 milligravities	Type 2 diabetes	0.504(0.305–0.833)	0.007	-	-	-	-
fraction accelerations > 425 milligravities	Fasting insulin	0.948(0.803–1.120)	0.536	1.036	0.595	-0.074	0.631

Regarding accelerometer-assessed fraction accelerations of > 425 milligravities, a significant association was found between a 1-SD-increase in such accelerations and a 0.22-lower BMI, a 0.154-mmol/L decrease in serum triglycerides, and a 50% reduced risk of T2D.

To summarize, the research findings indicate that metabolic disorders potentially contribute to the vulnerability to depression associated with leisure sedentary behavior, as evidenced by the risk factor analyses conducted.

## Discussion

This study utilized public psychiatric disorder GWAS data on a large scale to investigate the potential causal connection between depression, schizophrenia, bipolar disorder and leisure sedentary behavior/physical activity. Various MR methods were employed to analyze this relationship. The findings indicate that physical activity, as measured by accelerometer-assessed fraction accelerations of > 425 milligravities, can lower the likelihood of depression, while casual TV watching can increase the risk. Considering the substantial prevalence and detrimental consequences of depression, it is imperative to explore strategies for improving lifestyles in view of preventing this condition.

With the development of the social economy, people’s lifestyles have changed a great deal, with many including a reduction in sports time and an increase in leisure sedentary behavior time. These unhealthy lifestyles seriously affect people’s physical health, as well as their spiritual world. It is crucial to acknowledge that a persistent lack of physical activity and increased sedentary behavior are frequently linked to adverse physical and mental health outcomes, along with an elevated susceptibility to various chronic diseases, such as obesity, T2D, cardiovascular disease, and cancer [29–32]. Importantly, studies have indicated that detrimental lifestyle choices, insufficient physical activity, and obesity are associated with an elevated likelihood of developing depression [33–35]. Notwithstanding the findings from limited observational studies, there is currently insufficient direct evidence to establish a causal relationship between sedentary behavior and depression. In contrast to unrealistic large-scale prospective clinical trials, due to the need for long-term observation, MR studies have revealed the fact that leisure sedentary behavior/physical activity is associated with responding to depression in a time- and cost-effective manner.

The present study further confirms that, from a causal perspective, the risk of depression is increased by sedentary behavior. Several factors can be attributed to the possible causes of depression resulting from this type of behavior. Sedentary behavior often involves prolonged periods of sitting or lying down, which leads to reduced physical activity. Studies have shown that regular exercise and physical activity can improve mental health and decrease the risk of depression [36, 37]. Decreased social interaction and increased isolation can be associated with sedentary behavior. Devoting an excessive amount of time to sedentary activities, such as watching television or engaging in video game activities, can potentially restrict the opportunities for social interaction, thereby contributing to emotions of loneliness and isolation [38, 39]. Sedentary behavior, especially late-night use of electronic devices, can also disrupt sleep patterns, with insufficient or poor-quality sleep closely linked to an increased risk of depression [40]. Such activities can negatively affect sleep by disrupting the circadian rhythm, leading to mood disturbances and depressive symptoms [40]. Additionally, many sedentary activities are indoor-based, resulting in limited exposure to natural light. Sunlight exposure has a positive impact on mental health and is associated with the production of serotonin, a neurotransmitter that regulates mood. Reduced access to natural light due to sedentary behavior may contribute to the development or worsening of depression [41, 42]. The present study also found that there was a decrease in the risk of depression when fraction accelerations of > 425 milligravities were assessed using an accelerometer. A previous study found that accelerometer-based activity reduces the risk of major depressive disorder [43]. Growing evidence suggests that lifestyle behaviors influence disease development by altering epigenetic processes [44, 45]. It has been found that 6 months of exercise intervention can change the methylation of DNA associated with obesity and diabetes [46]. Therefore, exercise may also reduce the risk of depression through epigenetic effects, and the specific mechanism needs to be further studied. Therefore, reducing sedentary behavior, especially the time spent watching TV, while increasing physical activity (fraction accelerations of > 425 milligravities) is crucial to the prevention and treatment of depression.

Furthermore, our comprehensive analyses revealed numerous potential reasons for this causal link between depression and watching television. Initially, various indicators related to obesity were identified, such as BMI, triglycerides, fasting insulin, and T2D, which could potentially function as mediators linking television viewing and the occurrence of depression. Substantial evidence suggests that chronic low-grade inflammation in the body is correlated with metabolic disorders such as obesity and diabetes. Inflammation has the capability to impact the brain and neurotransmitter levels, potentially contributing to the development of depression. The overproduction of pro-inflammatory cytokines, such as tumor necrosis factor-alpha and interleukin-6, has been observed in cases of depression [47, 48]. Additionally, metabolic diseases have the potential to disrupt the hypothalamic-pituitary-adrenal (HPA) axis, resulting in the dysregulation of stress response and cortisol production. The chronic activation of the HPA axis and abnormal cortisol levels have been linked to depression [49]. When compared with computer usage and driving, watching television is a form of leisure entertainment that is more immersive and less reflective, with a limited scope for communication. Consistently, prolonged television viewing has been associated with poor physical and mental health, which could also partly contribute to depression. It should be emphasized that additional research is imperative for establishing the exact degree to which sedentary behavior mediation is implicated in depression. Without specific mediation analysis, it is not possible to determine the direct impact of television watching on depression.

In addition, our study also found that driving has a slight effect in reducing the risk of schizophrenia, while leisure television viewing has a significant protective effect against the onset of bipolar disorder. This is an interesting finding, although observational studies have not yet reported this phenomenon. This also suggests that the effects of watching TV on different psychiatric disorders are different, and different situations need to be treated differently. On the other hand, there is no apparent association between physical activity, computer use, and the onset of schizophrenia or bipolar disorder.

The two-sample MR analysis method has several merits. First, in contrast to conventional observational studies, employing MR methods permits the estimation of effects with greater reliability and minimizes the potential for confounding and reverse causation. Second, the inclusion of individuals with European ancestry in the data analysis greatly reduces the potential impact of population stratification. Finally, the implementation of rigorous procedures effectively curtails the deviation attributable to instrumental variables.

Nevertheless, this study has certain limitations. First, there is insufficient personal information in the stratified secondary data to analyze gender and age. Second, the MR analysis postulates a linear correlation between exposure and outcome effects. However, such an assumption impedes the assessment of the non-linear correlation between depression risk and sedentary behavior and physical activity. Third, since the study was limited to individuals of European descent, the generalizability of the present findings to other ethnic populations is unfeasible. Fourth, subgroup analysis of different depression types was not feasible due to a lack of relevant information.

In summary, the present study involving two-sample MR presents compelling findings supporting the genetic underpinnings of the association between heightened sedentary behavior, reduced physical activity, and depression. As a result, these novel findings underscore the importance of reducing television watching time and increasing physical activity as preventive measures for depression. In addition, our study also found that leisure television viewing has a significant protective effect against the onset of bipolar disorder. This suggests that the effects of watching TV on different psychiatric disorders are different, and different situations need to be treated differently.

### Electronic supplementary material

Below is the link to the electronic supplementary material.

Supplementary Material 1

Supplementary Material 2

## Data Availability

The datasets used and/or analysed during the current study are available from the corresponding author on reasonable request.
