# Supplementary material for: Causal links between sedentary behavior, physical activity, and psychiatric disorders: a Mendelian randomization study
Source: Ann Gen Psychiatry. 2024 Feb 29;23:9. doi: 10.1186/s12991-024-00495-0 (PMC10905777; doi:10.1186/s12991-024-00495-0)
Supplement: Supplementary file 1 — Supplementary Material 1 [file 12991_2024_495_MOESM1_ESM.pdf]

Additional file 1: Table S1. Instrument variables of television watching

SNP	effect_allel	other_allel	beta	eaf	se	p
rs1004172	T	C	0.0180931	0.807695	0.00273852	3.92E-11
rs1005432	G	A	0.0172449	0.576024	0.00219021	3.45E-15
rs1014559	C	G	-0.014845	0.409418	0.00220802	1.78E-11
rs1018985	A	G	-0.020463	0.56785	0.00217999	6.19E-21
rs1031423	T	C	-0.018519	0.215208	0.00262613	1.76E-12
rs1073762	T	A	0.0144141	0.273668	0.00241921	2.55E-09
rs1077174	C	T	-0.014282	0.716605	0.00239463	2.46E-09
rs1077264	C	T	0.0247995	0.107916	0.00349653	1.32E-12
rs1087686	G	A	-0.01334	0.427197	0.0021844	1.01E-09
rs1093283	C	T	-0.013106	0.494411	0.00216152	1.34E-09
rs1113079	C	T	0.0129223	0.601737	0.00221055	5.04E-09
rs1119010	G	T	-0.01708	0.817212	0.00284699	1.98E-09
rs1124548	T	C	-0.013233	0.61325	0.00222132	2.57E-09
rs1146002	G	C	-0.016243	0.788982	0.00264223	7.88E-10
rs1168919	A	G	0.0184644	0.597665	0.00220443	5.47E-17
rs1171433	G	A	0.0144017	0.569041	0.00218888	4.72E-11
rs1181010	A	T	0.0162518	0.701438	0.00235696	5.38E-12
rs1210570	C	T	-0.012883	0.395851	0.00220843	5.43E-09
rs1243182	C	T	-0.018592	0.689639	0.00234162	2.03E-15
rs1249150	G	A	-0.014261	0.670306	0.00229906	5.55E-10
rs1255451	T	C	0.0207024	0.584071	0.00219328	3.76E-21
rs1310732	C	T	-0.029171	0.925644	0.00412256	1.49E-12
rs1720789	G	A	0.015694	0.657407	0.00228666	6.73E-12
rs1772747	C	T	0.017551	0.832295	0.00296085	3.07E-09
rs1778921	T	C	0.0185965	0.75605	0.00251401	1.39E-13
rs2034768	A	G	0.0147356	0.48743	0.00215816	8.62E-12
rs2045147	A	G	0.0126686	0.449407	0.00217659	5.87E-09
rs2073869	C	T	0.0186112	0.833626	0.0029076	1.54E-10
rs2173650	G	T	0.017845	0.852206	0.00304387	4.56E-09
rs2184364	A	G	0.0156396	0.782382	0.00263677	3.00E-09
rs2447098	C	A	-0.014947	0.475175	0.00217845	6.82E-12
rs2460	G	A	-0.015294	0.736281	0.00245865	4.96E-10
rs2616830	G	A	0.0164651	0.461988	0.00216551	2.89E-14
rs262890	A	G	-0.018568	0.698905	0.00235563	3.21E-15
rs303753	G	A	-0.01446	0.652948	0.00229051	2.74E-10
rs3481147	G	A	0.0152829	0.767813	0.00255709	2.28E-09
rs3486402	A	G	-0.026382	0.933558	0.00435706	1.41E-09
rs374722	G	A	0.0244932	0.150476	0.0030238	5.49E-16
rs3796386	G	A	-0.026171	0.571888	0.0021795	3.23E-33
rs42210	G	C	-0.013852	0.289169	0.00239536	7.35E-09
rs4382592	T	G	0.0136721	0.301425	0.00235776	6.68E-09
rs4577309	A	G	0.0160037	0.468698	0.00216796	1.56E-13
rs4845364	A	G	-0.015301	0.495003	0.00215604	1.28E-12
rs4973576	C	A	-0.014523	0.298072	0.00237947	1.04E-09
rs5610324	C	T	0.0297647	0.943942	0.00475247	3.78E-10
rs5685876	G	A	-0.014879	0.700762	0.00236956	3.40E-10
rs5758521	T	G	-0.016733	0.826832	0.00285006	4.33E-09
rs6131281	C	T	0.0160808	0.596509	0.00220553	3.07E-13
rs6141814	C	A	-0.013502	0.613455	0.00222588	1.31E-09
rs6237937	G	T	-0.026025	0.928551	0.0042322	7.79E-10
rs6264163	A	G	0.0144237	0.691839	0.00233911	6.99E-10
rs6472942	T	C	-0.013163	0.568486	0.00218761	1.77E-09
rs6673341	T	G	-0.014513	0.465333	0.0021693	2.23E-11
rs6797840	A	C	-0.016103	0.455899	0.00218412	1.67E-13
rs6825241	C	A	-0.016958	0.536204	0.00216609	4.92E-15
rs6850494	A	C	-0.01433	0.615571	0.00222236	1.13E-10

rs6905544	A	G	-0.018994	0.398622	0.00221031	8.46E-18
rs7184800	G	A	0.0168167	0.696784	0.00234935	8.18E-13
rs7189927	T	C	0.0149741	0.355786	0.00225865	3.36E-11
rs7248205	C	T	0.0139253	0.397614	0.00221689	3.35E-10
rs7278169	G	A	-0.01868	0.797367	0.00267802	3.05E-12
rs7282889	C	T	0.0192918	0.869256	0.00329837	4.95E-09
rs7283469	G	A	0.0226559	0.858074	0.0031007	2.74E-13
rs749671	G	A	0.015654	0.62863	0.00223821	2.67E-12
rs7564130	T	C	-0.014986	0.640601	0.00225071	2.77E-11
rs7693082	G	C	0.0150716	0.299686	0.00235845	1.65E-10
rs7693703	G	A	0.0228437	0.910033	0.0038412	2.73E-09
rs7834121	G	T	-0.01396	0.504369	0.00216371	1.11E-10
rs7991062	C	G	-0.017738	0.658663	0.00228727	8.82E-15
rs801733	A	C	0.0168585	0.641215	0.00225323	7.32E-14
rs8756	C	A	-0.01345	0.484619	0.00216618	5.33E-10
rs9471333	C	T	0.0131038	0.450187	0.00216935	1.54E-09
rs9563168	G	A	0.0175868	0.791482	0.00266741	4.30E-11
rs9569734	A	G	0.0188846	0.843521	0.00300692	3.38E-10
rs9718104	T	G	-0.040768	0.94155	0.00461017	9.31E-19
rs9834970	T	C	0.0127684	0.501031	0.00215775	3.27E-09
rs9867121	C	A	0.0194994	0.817728	0.00280905	3.88E-12
rs9902312	T	C	0.0153237	0.683148	0.00232706	4.55E-11

Additional file 1: Table S2. Instrument variables of computer used

SNP	effect_allele	other_allele	eaf	beta	se	p
rs10208088	C	T	0.418634	0.0131026	0.00223225	4.37E-09
rs113851275	G	A	0.891576	-0.0209442	0.00353742	3.20E-09
rs1448355	C	T	0.381282	-0.0146118	0.00227388	1.31E-10
rs166835	C	T	0.443342	0.0131356	0.00222179	3.38E-09
rs206965	T	C	0.206729	0.0156007	0.00271472	9.10E-09
rs2345941	A	G	0.550313	0.014647	0.00221726	3.95E-11
rs2748985	T	C	0.454667	-0.0153444	0.00221274	4.07E-12
rs35933007	G	A	0.771615	-0.0151917	0.00264445	9.21E-09
rs4073003	A	G	0.873541	0.0201634	0.00331978	1.25E-09
rs4977839	G	A	0.584465	-0.0199181	0.00223302	4.67E-19
rs55772938	A	G	0.704749	-0.0151223	0.00241703	3.94E-10
rs6744254	C	T	0.47126	-0.0159012	0.00220373	5.37E-13
rs6774533	C	T	0.294245	-0.0149436	0.00245831	1.21E-09
rs73578186	C	T	0.675347	0.0149904	0.00236483	2.31E-10
rs9372625	G	A	0.61853	-0.0183911	0.00227197	5.74E-16

Additional file 1: Table S3. Instrument variables of driving

SNP	effect_allel	other_allel	eaf	beta	se	p
rs10186876	A	G	0.360301	0.014405	0.002338	7.20E-10

Additional file 1: Table S4. Instrument variables of MVPA

SNP	effect_allele	other_allele	eaf	beta	se	P_value
rs10145335	G	A	0.749	-0.014	3.00E-03	3.06E-06
rs1043595	G	A	0.717	0.014	2.00E-03	2.56E-12
rs1186721	G	A	0.684	-0.013	2.00E-03	8.03E-11
rs12912808	C	T	0.851	0.018	3.00E-03	1.97E-09
rs1921981	G	A	0.674	0.013	2.00E-03	8.03E-11
rs1974771	G	A	0.9	-0.021	4.00E-03	1.52E-07
rs2035562	A	G	0.328	-0.014	2.00E-03	2.56E-12
rs2114286	A	G	0.466	-0.012	2.00E-03	1.97E-09
rs2942127	G	A	0.175	0.016	3.00E-03	9.64E-08
rs2988004	T	G	0.558	-0.013	2.00E-03	8.03E-11
rs77742115	T	C	0.862	-0.018	3.00E-03	1.97E-09
rs7804463	T	C	0.53	0.015	2.00E-03	6.38E-14
rs877483	T	C	0.433	0.012	2.00E-03	1.97E-09
rs921915	T	C	0.412	-0.014	2.00E-03	2.56E-12

Additional file 1: Table S5. Instrument variables of accelerations assessed physical activity

SNP	effect_allele	other_allele	eaf	beta	se	P_value
rs3451743	C	A	0.879	0.308	5.60E-02	4.40E-08
rs6775319	A	T	0.271	0.225	4.10E-02	3.50E-08
rs9293503	T	C	0.888	0.329	5.90E-02	2.10E-08
rs1252226	G	A	0.657	0.211	3.80E-02	3.90E-08
rs1101273	A	G	0.668	0.225	3.90E-02	5.40E-09
rs1481932	A	C	0.957	-0.51	9.20E-02	3.10E-08
rs5949965	A	T	0.656	-0.228	3.80E-02	2.40E-09

Additional file 1: Table S6. Instrument variables of fraction accelerations > 425 milli-gravities

SNP	effect_allel	other_allel	eaf	beta	se	P_value
rs1856329	A	C	0.801	0.027	5.00E-03	9.00E-08
rs6433478	T	C	0.457	-0.024	4.00E-03	1.20E-08
rs6244362	T	C	0.767	-0.026	5.00E-03	1.40E-07
rs7263336	G	A	0.711	-0.023	5.00E-03	4.10E-07
rs4754194	C	T	0.773	-0.025	5.00E-03	2.40E-07
rs743580	A	G	0.51	0.025	4.00E-03	1.30E-09
rs1668835	T	A	0.688	-0.023	0.004	3.10E-07

Additional file 1: Table S7.MR estimates of the causal association between physical activity and leisure sedentary behaviors and the risk of depression.

Exposure	Methods	Outcome	
		OR (95% CI)	*P*
MVPA	IVW	0.963(0.917-1.010)	0.128
	MR-Egger	0.714(0.510-1.001)	0.074
	WMS	0.948(0.884-1.015)	0.129
	Simple mode	0.940(0.830-1.064)	0.351
	Weighted mode	0.939(0.826-1.068)	0.358
Accelerometer assessed PA	IVW	0.996(0.992-1.000)	0.089
	MR-Egger	0.991(0.971-1.010)	0.422
	WMS	0.996(1.991-1.002)	0.308
	Simple mode	0.998(0.988-1.007)	0.75
	Weighted mode	0.998(0.990-1.007)	0.792
Accelerometer fraction of accelerations>425	IVW	0.951(0.914-0.989)	0.013
	MR-Egger	0.705(0.338-1.467)	0.393
	WMS	0.960(0.914-1.009)	0.114
	Simple mode	0.977(0.907-1.053)	0.573
	Weighted mode	0.980(0.910-1.055)	0.613
Television watching	IVW	1.027(1.001-1.053)	0.04
	MR-Egger	1.054(0.930-1.194)	0.407
	WMS	1.003(0.975-1.033)	0.788
	Simple mode	0.979(0.907-1.057)	0.595
	Weighted mode	1.004(0.979-1.030)	0.585
Computer use	IVW	1.031(0.974-1.092)	0.283
	MR-Egger	1.083(0.704-1.667)	0.722
	WMS	1.029(0.996-1.096)	0.37
	Simple mode	1.118(0.979-1.278)	0.121
	Weighted mode	1.022(0.922-1.133)	0.677
	MR-PRESSO	0.95 (0.84-1.08)	0.45
Driving	Wald ratio	1.015(0.858-1.200)	0.59

MR, Mendelian randomization; MRE-IVW, multiplicative random effects-inverse variance weighted; WM, weighted median; PA: physical activity.

Additional file 1: Table S8.MR estimates of the causal association between physical activity and leisure sedentary behaviors and the risk of schizophrenia.

**Exposure**	**Methods**	**Outcome**	
		OR (95% CI)	*P*
MVPA	IVW	1.435(0.737-2.795)	0.289
	MR-Egger	4.729(0.045-499.927)	0.527
	WMS	1.128(0.565-2.250)	0.733
	Simple mode	4.184(0.560-31.249)	0.188
	Weighted mode	4.066(0.707-23.374)	0.142
Accelerometer assessed PA	IVW	1.027(0.980-1.076)	0.268
	MR-Egger	0.981(0.794-1.213)	0.87
	WMS	1.030(0.983-1.080)	0.212
	Simple mode	1.032(0.966-1.107)	0.416
	Weighted mode	1.032(0.966-1.103)	0.393
Accelerometer fraction of accelerations>425 milli-gravities	IVW	1.052(0.708-1.564)	0.802
	MR-Egger	0.004(0.000-2.501)	0.154
	WMS	1.089(0.724-1.638)	0.684
	Simple mode	1.020(0.589-1.766)	0.947
	Weighted mode	1.032(0.966-1.103)	0.917
Television watching	IVW	0.912(0.726-1.145)	0.428
	MR-Egger	0.730(0.237-2.244)	0.585
	WMS	0.835(0.654-1.067)	0.149
	Simple mode	0.488(0.224-1.062)	0.075
	Weighted mode	0.600(0.294-1.222)	0.164
Computer use	IVW	0.875(0.548-1.397)	0.575
	MR-Egger	3.946(0.082-189.837)	0.499
	WMS	1.061(0.645-1.747)	0.815
	Simple mode	1.712(0.560-5.243)	0.362
	Weighted mode	1.832(0.556-6.038)	0.336
Driving	Wald ratio	0.092(0.010-0.827)	0.033

MR, Mendelian randomization; MRE-IVW, multiplicative random effects-inverse variance weighted; WM, weighted median; PA: physical activity.

Additional file 1: Table S9.MR estimates of the causal association between physical activity and leisure sedentary behaviors and the risk of bipolar disorder.

**Exposure**	**Methods**	**Outcome**	
		OR (95% CI)	*P*
MVPA	IVW	1.242(0.816-1.889)	0.312
	MR-Egger	4.530(0.220-93.380)	0.347
	WMS	1.237(0.719-2.129)	0.277
	Simple mode	1.321(0.484-3.606)	0.596
	Weighted mode	1.268(0.517-3.107)	0.613
Accelerometer assessed PA	IVW	1.032(0.974-1.093)	0.284
	MR-Egger	0.920(0.782-1.081)	0.495
	WMS	1.035(0.965-1.110)	0.333
	Simple mode	1.046(0.958-1.143)	0.418
	Weighted mode	1.045(0.953-1.146)	0.447
Accelerometer fraction of accelerations >425 milli-gravities	IVW	1.014(0.614-1.674)	0.956
	MR-Egger	0.426(0.000-899.758)	0.863
	WMS	0.984(0.540-1.792)	0.957
	Simple mode	0.965(0.500-1.863)	0.926
	Weighted mode	0.966(0.484-1.929)	0.931
Television watching	IVW	0.719(0.567-0.912)	0.006
	MR-Egger	0.219(0.066-0.728)	0.017
	WMS	0.833(0.632-1.098)	0.195
	Simple mode	0.428(0.188-0.976)	0.049
	Weighted mode	0.991(0.479-2.051)	0.982
Computer use	IVW	1.315(0.903-1.916)	0.153
	MR-Egger	15.665(0.621-395.076)	0.119
	WMS	1.513(0.919-2.494)	0.104
	Simple mode	1.897(0.707-5.088)	0.224
	Weighted mode	1.936(0.727-5.154)	0.207
Driving	IVW	0.882(0.212-3.679)	0.863
	MR-Egger	11.124(0.000-12512433)	0.767
	WMS	0.972(0.395-2.391)	0.951
	Simple mode	1.088(0.261-4.544)	0.915
	Weighted mode	1.089(0.320-3.703)	0.901

MR, Mendelian randomization; MRE-IVW, multiplicative random effects-inverse variance weighted; WM, weighted median; PA: physical activity.
