# Supplementary material for: Causal links between sedentary behavior, physical activity, and psychiatric disorders: a Mendelian randomization study
Source: Ann Gen Psychiatry. 2024 Feb 29;23:9. doi: 10.1186/s12991-024-00495-0 (PMC10905777; doi:10.1186/s12991-024-00495-0)

**Additional file 2**: Supplementary figures.

Figure S1. (a) Forest plot and (b) and leave-one-out (LOO) analysis results for the impact of leisure television watching on depression.


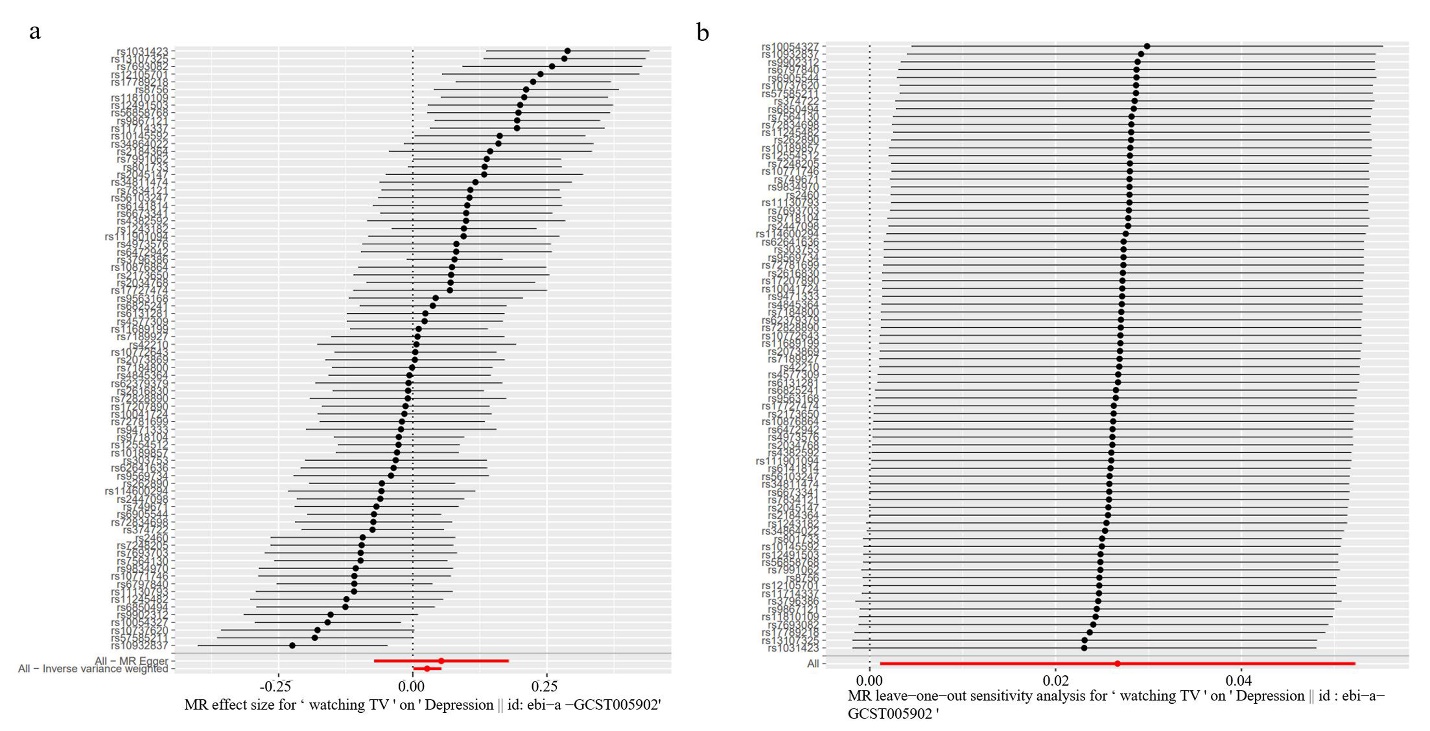


Figure S2. (a) Forest plot and (b) LOO analysis results for the impact of fraction accelerations of >425 milligravities on depression.


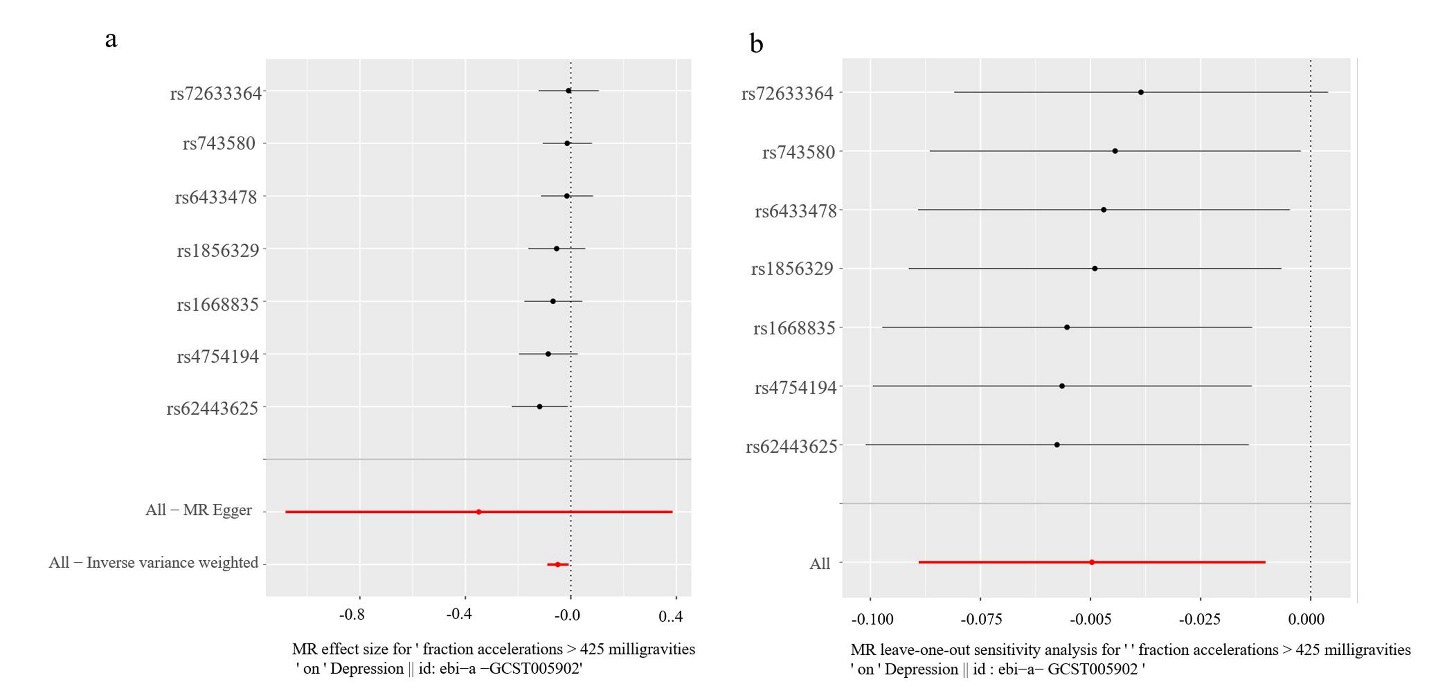


Figure S3. Forest plot analysis results for the impact of driving on schizophrenia.


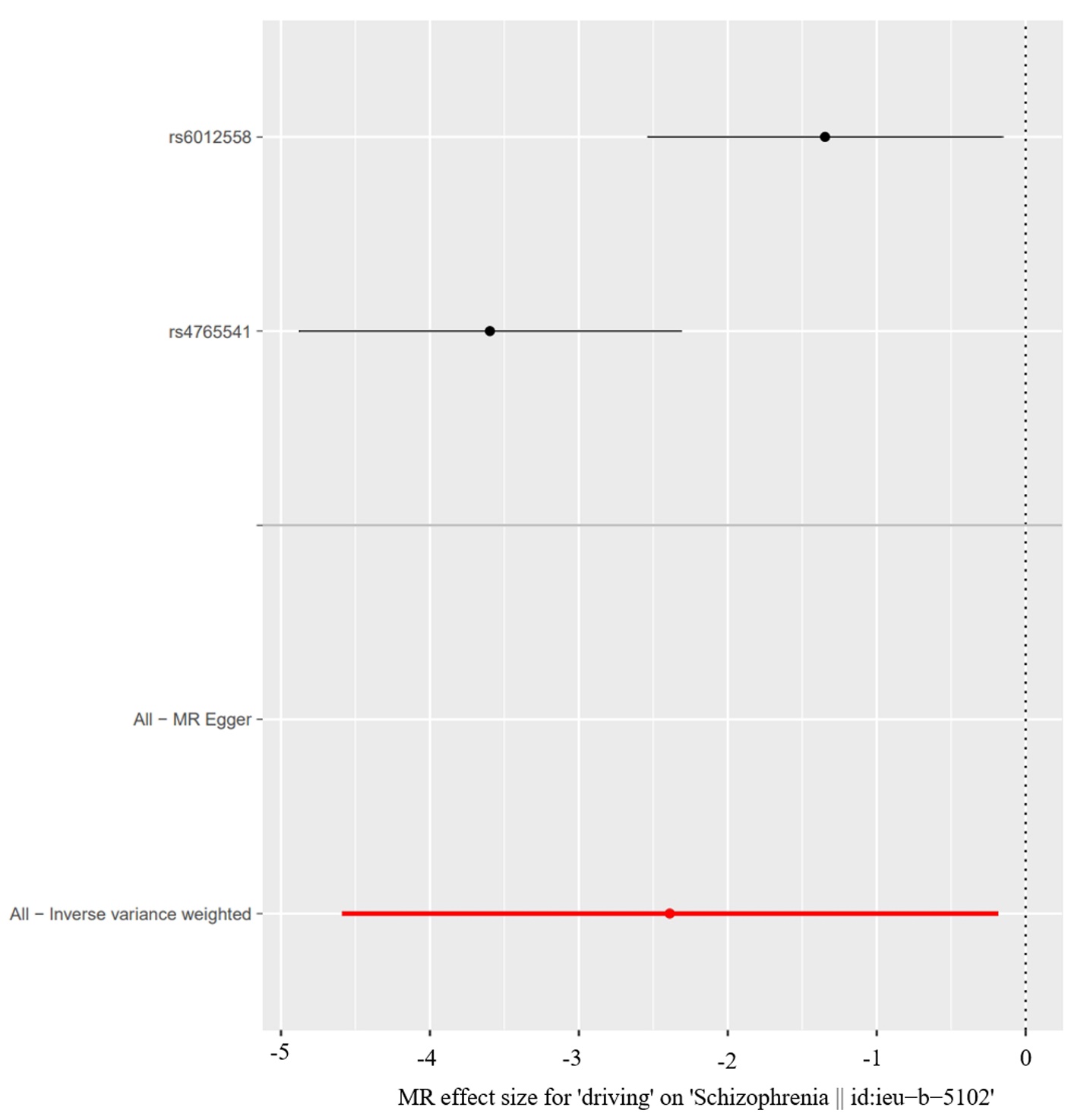


Figure S4. (a) Forest plot and (b) LOO analysis results for the impact of television watching on bipolar disorder.


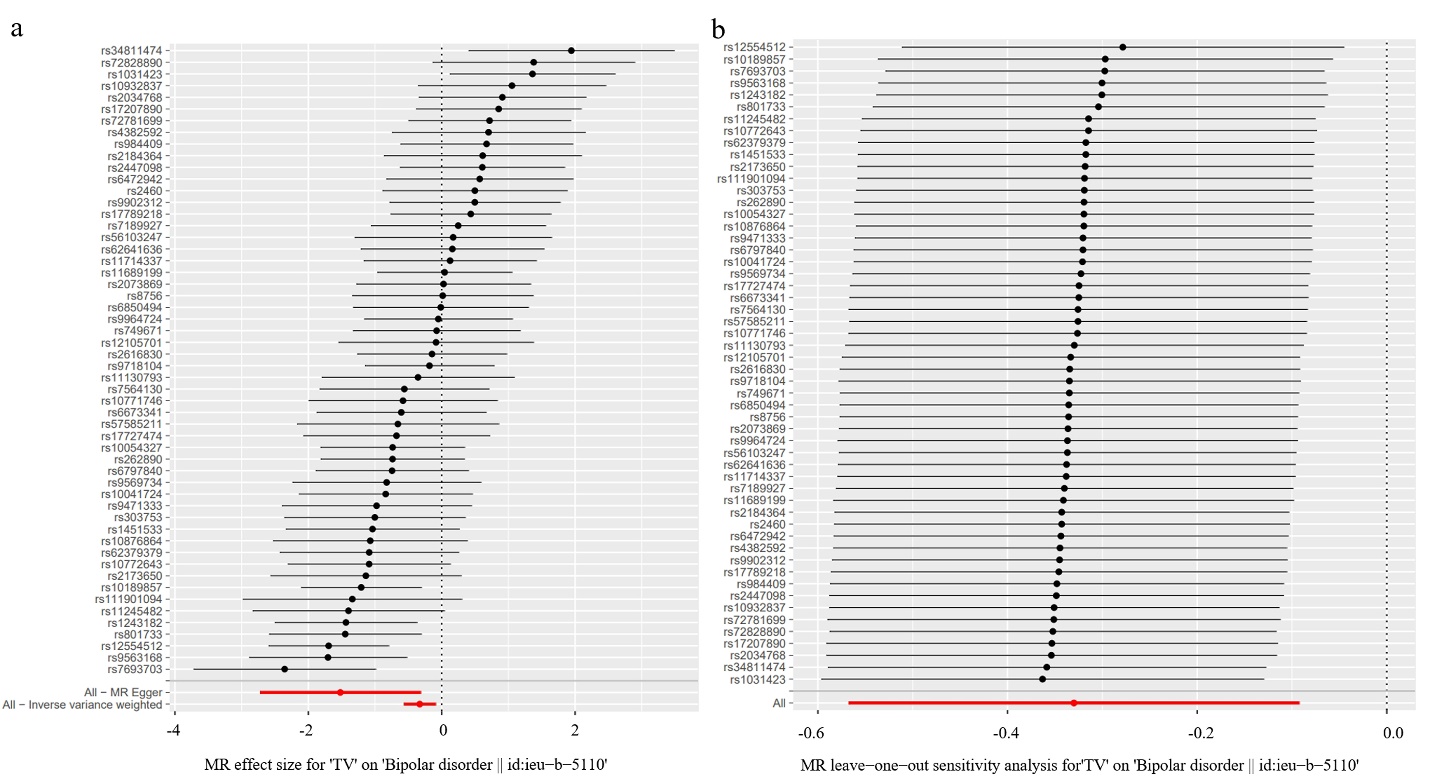

Supplement: Supplementary file 2 — Supplementary Material 2 [file 12991_2024_495_MOESM2_ESM.docx]
